# How to prevent 3 million deaths worldwide: a systematic review of occupational accident research—a factor- and cost-based approach

**DOI:** 10.1093/eurpub/ckae197

**Published:** 2024-12-04

**Authors:** Rosa María Cañaveras Perea, Ángel Tejada Ponce, María Pilar Sánchez González

**Affiliations:** Department of Business Administration, Faculty of Economic and Business Sciences, University of Castilla-La Mancha, Albacete, Spain; Department of Business Administration, Faculty of Economic and Business Sciences, University of Castilla-La Mancha, Albacete, Spain; Department of Business Administration, Faculty of Economic and Business Sciences, University of Castilla-La Mancha, Albacete, Spain

## Abstract

Occupational accidents have emerged as a global concern, necessitating a comprehensive examination of their determinants and associated costs. This review aims to summarize, synthesize, and organize the factors and cost drivers of occupational accidents, exploring whether there is a gender perspective. Adhering to PRISMA guidelines, we performed a narrative synthesis to systematically review relevant literature. A systematic search was conducted in the electronic databases PubMed, Web of Science, and Scopus. Two researchers screened all records to eliminate any duplicates, and they selected the articles for full review. A third researcher was consulted to resolve discrepancies and reach a consensus. The analysis of 15 studies revealed diverse perspectives; in terms of determinants, studies on organizational aspects and the theory of human error were grouped together, while in cost drivers, the human capital model and willingness to pay were the most frequently used. Gender, meanwhile, is identified as a determinant variable for accident rate. Additionally, limitations such as data underestimation were noted in the existing literature. The review highlights the need for empirical studies capable of addressing both determinants and cost drivers. It also provides guidelines for researchers to design studies that are more comparable across different contexts, including the gender debate.

## Introduction

Within the framework of the 2030 Sustainable Development Goals (SDGs), two key targets related to occupational health are emphasized: Good health and well-being (SDG 3), and Decent work for all (SDG 8). However, each year, exposure to occupational hazards in nearly 3 million fatalities [1]. These incidents place a significant strain on healthcare systems and reduce productivity, leading to substantial costs that go far beyond ‘mere’ business and hospital expenses, affecting society as a whole. To effectively address this public health issue, it is essential to understand its underlying determinants and their economic valuation. There is growing advocacy for integrating a gender perspective into various aspects of occupational accidents [2]. Nevertheless, these approaches remain relatively scarce and are seldom evaluated in conjunction. Therefore, the main objective of this review is to summarize, synthesize, and organize the patterns in the literature on these three approaches, analysing and categorizing the conclusions and limitations reported. Additionally, this review seeks to provide valuable information and scientific evidence for health professionals, researchers, and policymakers.

## Methods

### Search strategy

The guidelines outlined in the PRISMA statement for reporting systematic reviews and meta-analyses [3], along with those from the Joanna Briggs Institute (JBI) [4], were followed when conducting this systematic review. The literature search was conducted in November 2023, encompassing the entire available publication period for a comprehensive analysis. Given the multidisciplinary nature of the review, three databases were consulted: PubMed, Web of Science, and Scopus. Table 1 displays the search strategy and the terms included, as well as the exclusion and inclusion criteria.

**Table 1.** Search strategy

Category	Criteria	Details
Keywords	Inclusion	(‘occupational accidents’ OR ‘occupational injuries’ OR ‘accidents at work’ OR ‘workplace accidents’ OR ‘workplace injuries’) AND (‘determinants’ OR ‘factors’ OR ‘cost’ OR ‘expenditure’ OR ‘gender’ OR ‘sex’)
	Exclusion	NOT (‘ergonomics’ OR ‘epidemiologic’ OR ‘epidemiology’ OR ‘epidemiological’ OR ‘control’ OR ‘surveillance’ OR ‘unions’ OR ‘system’ OR ‘protocol’ OR ‘safety culture’ OR ‘fall’ OR ‘falls’ OR ‘falling’ OR ‘traumatic’ OR ‘heart’ OR ‘eye’ OR ‘musculoskeletal’ OR ‘back’ OR ‘neck’ OR ‘arm’ OR ‘elbow’ OR ‘hand’ OR ‘foot’ OR ‘time lost’ OR ‘HIV’ OR ‘rehabilitation’)
Inclusion criteria	Language	Only English
	Article type	Original research articles
	Focus	Study of determinants and costs of occupational accidents, particularly related to gender or sex
Exclusion Criteria	(i) Body parts	Focus on specific body part injuries
	(ii) Factors/costs	Lack of information on explanatory factors or costs
	(iii) Ergonomics	Solely focused on ergonomics or rehabilitation
	(iv) Safety issues	Focus only on safety environment issues
Further Discarding	(a) Global accidents	Focused on a single globally known accident
	(b) Specific sectors	Concentrated on injuries within specific sectors or professions
	(c) Single determinants	Focused solely on one determinant, excessively specific cases, or did not meet inclusion criteria for study design
	(d) Descriptive Studies	Excluded purely descriptive articles lacking statistical methodologies
	(e) Related topics	Did not directly address the chosen topic of the review or solely focused on study design

We understand determinant as a collective or individual risk factor (or set of factors) that is causally related to a health condition, outcome, or other defined characteristic [5].

### Study selection

**Figure 1.**
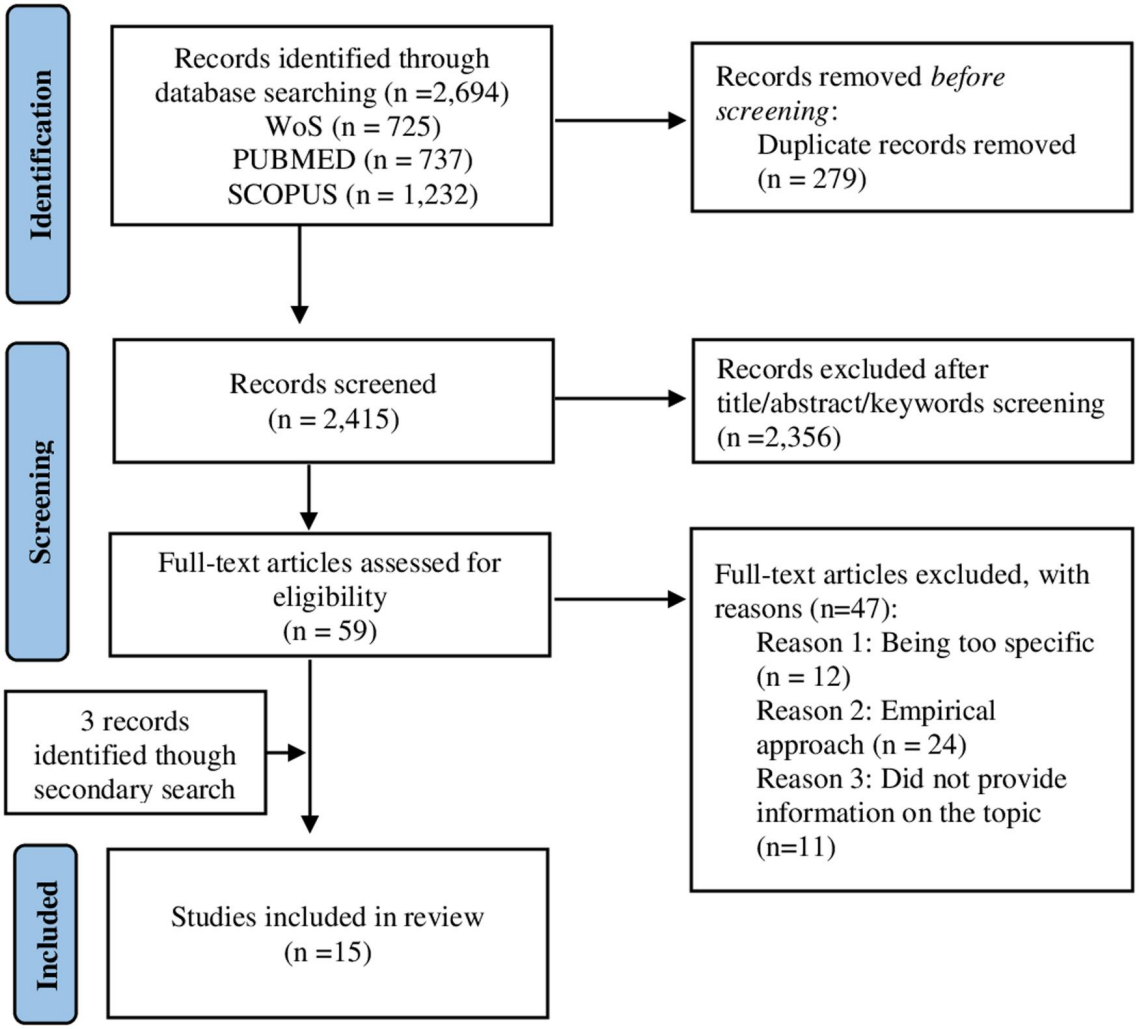
PRISMA flowchart.

The study selection and data extraction process consisted of three phases (see Fig. 1). First, two researchers (R.M.C.P. and Á.T.P.) initially screened all records to eliminate duplicates. Next, both researchers independently reviewed the titles, abstracts and keywords of the 2415 studies retrieved. They selected only complete articles. At this stage, the results were compared, and a third researcher (M.P.S.G.) was consulted to resolve possible discrepancies in the inclusion criteria and to reach an objective consensus. In the third phase, the two researchers (R.M.C.P. and Á.T.P.) read all the articles selected after applying the inclusion and exclusion criteria. The full text of 59 articles was reviewed. Of these, 47 were excluded based on the exclusion criteria. Again, the third researcher (M.P.S.G.) was involved in resolving any doubts or conflicts. To ensure no relevant studies were missed in the initial search, the references of the 12 accepted articles were reviewed, resulting in the identification of 3 additional studies. Thus, a total of 15 articles were included in the review.

## Results

Due to the broad range of knowledge areas and the varied aspects of occupational accidents addressed in the review articles, the resulting data for analysis are inherently heterogeneous. Therefore, we chose for a narrative synthesis methodology to summarize the findings. A narrative approach, or qualitative systematic review, is suitable in this context, as meta-analysis of reported studies would be unsuitable given the wide heterogeneity of data.

The narrative synthesis of the literature highlights both commonalities and disparities, providing insights for future research in this field. The results are presented in tables to enhance comprehension of the studies, methodologies, and key findings (see Tables 2 and 3).

**Table 2.** Results of the systematic review: determinants

ID	Author	Geographical	Dates	Source	Method	Dependent variable	Determinants	Sex/gender as a factor?	Synthesis of results	Conclusion
1	Bhattacherjee *et al*. [6]	France (Lorraine)	May and July 1996	Primary: own survive	Loglinear model	Incidence of occupational injuries	**Demographic and individual factors:** - Sex. - Job. - Age. - Body mass index. - Smoking habit. - Excess alcohol use. - Regular psychotropic drug use. - Presence of a disease. **Work conditions and business factors:** - Previous injuries.	Yes	- Sex, youth, smoking, heavy alcohol consumption, overweight, use of psychotropic drugs and diseases influenced occupational injuries. - Men younger individuals, smokers, heavy drinkers, regular psychotropic users, and those suffering from disease were at higher risk.	Preventive measures focusing on working conditions, risk assessment and job awareness should be target men, young workers, smokers, alcohol consumers, overweight workers and those with psychosomatic conditions.
2	Kirschenbaum *et al*. [7]	Israel	1998	Primary: own survive	Logistic regression and factor analysis	Accident proneness (first-time vs. repeat injured)	**Demographic and individual factors:** - Gender. - Age. - Religion. - Marital status. - Number of children. - Migration status. - Educational level. **Work conditions and business factors:** - Economic sector. - Occupation. - Length of service. - Monthly personal income. - Average working hours. - Type of employment. - Risk level.	Yes	- Gender and marital status predicted injury likelihood. - Work environment and emotional well-being affected injury risks.	- Workers emphasized inadequate safety conditions and excessive workload. - Subcontracting influenced accident proneness.
3	Chang *et al*. [8]	Taiwan	2002–14	Secondary: official sources	Regression method; Granger causality test; Kruskal-Wallis test.	Incidence rate of occupational injury insurance payments in various industries	**Economic factors**: - Business cycle indicators. - Composition of the labour force. **Work conditions and business factors:** - Average remuneration. - Labour relations. - Working conditions.	No	- Accidents, especially minor ones, were statistically significant linked to business cycles across several industries. - Working conditions affected accident rates; labour relations do not.	Security performance was also affected not only by endogenous factors but also by external factors, including economic fluctuations.
4	Davies *et al*. [9]	United Kingdom	1986–2005	Secondary: Official sources	Panel data analysis	Workplace injuries classified by severity	**Economic factors**: - Percentage of new hires. - Percentage of overtime. - Percentage of overtime working. - Work intensity ratio. - Percentage of manual labour. - Percentage of small business employment. - Percentage of temporary contracts. - Unemployment rate.	No	- Minor injuries are pro-cyclical, serious injuries are not affected by the level of economic activity. - Construction and manufacturing are affected by cyclical fluctuations. - Bargaining power and reporting behaviour are related to minor injury rates.	- Policies should improve new worker training, monitor unskilled workers’ integration, and set limits on productivity increases during economic expansions. - Fair injury reporting practices are needed to ensure consistent reporting across economic cycle.
5	Łyszczarz and Nojszewska [10]	Poland (66 sub-regions)	2002–14	Secondary: official sources	Panel data regression	(a) Total occupational accident rates per 1000 workers. (b) Accident rates among men and women separately per 1000 workers. (c) Days of work disability due to occupational accidents per employee	**Economic factors:** - Gross Domestic Product per capita. - Average earnings. - Unemployment rate. - Construction permits. **Work conditions and business factors:** - Employment in hazardous conditions. - Company size.	Yes	- Occupational accidents in Poland showed procyclical behaviour (more accidents during economic expansions). - A tighter relationship between economic situation and occupational accidents was found for men and the general population. - Women’s relationship was not always significant, as they often crisis-resistant sectors. - Men had a higher exposure, and there were also differences in the coefficient of temporal trend. - A higher percentage of medium-sized companies correlated with lower accident rate.	During periods of increased labour intensity, employers should focus on providing training and supervision for inexperienced workers and ensure experienced employees have sufficient recovery time.

**Table 3.** Results of the systematic review: cost drivers

Id	Author	Geographical	Dates	Source	Economic cost model?	Cost drivers	Currency	Synthesis of results	Conclusions
6	Leigh *et al*. [11]	United States	1993	Secondary: official sources	Willigness to pay	Medical cost Wage loss Household production loss Pain and suffering loss	USD	Fatal accidents account for one-fifth of total costs ($77 billion). Highest costs are among workers aged 17 and under; males incur higher costs than females; Agriculture, fishing and forestry had the highest cost per employed worker of all occupational groups and the highest percentage of costs as fatalities. Strains and sprains represented the greatest economic burden, accounting for more than one third of the total costs. The costs of pain and suffering were considerable and exceeded the benefits paid by workers' compensation.	Increasing costs influence debates on Occupational Safety and Health and workers’ compensation policies among stakeholders.
7	Kim [12]	South Korea	2013	Secondary: official sources	Willingness to pay	Loss of income Compensation payments Non-financial human costs Costs to maintain production Medical Cost Administrative costs	USD	- Total estimated costs of losses: US$27.224 billion (2.1% of the GDP of South Korea); individuals (79.5%) bear the most, followed by employers (20.4%) and government (0.1%). - Main costs: individuals focus on income loss; employers on compensation, government on national insurance and pension premiums.	- These costs facilitate the calculation of losses incurred due to industrial accidents at the corporate or governmental level. - The loss costs can be used by the government as basic data to establish industrial safety and health policies or to promote industrial safety awareness in Korea.
8	Thepaksorn and Pongpanich [13]	Thailand (Bangkok)	2008	Secondary: official sources	No	Medical cost Rehabilitation costs, Funeral compensation costs Compensation payments Loss of income	USD	- Occupational injuries and illnesses contributed to total health care costs and lost productivity in Bangkok. - Workers' compensation covered less than one half to one tenth of the costs of occupational injuries and illnesses compared to SSO payments for non-occupational injuries and illnesses ($246 million). - Non-fatal occupational injuries and illnesses total direct costs of $13.87 million: 71% were for medical care costs and 29% for compensation for lost earnings. - These costs excluded indirect costs related to pain and suffering or home health care.	There is a need to improve the quality of workers’ compensation healthcare services, including more occupational clinics and physicians in provinces.
9	Lebeau *et al*. [14]	Canada (Québec)	2005–07	Secondary: official sources	DALY, willingness to pay, and human capital	Medical costs Funeral costs Wage costs Administrative costs Pain and suffering costs Productivity losses	USD	- Estimated costs of occupational injuries/diseases in Quebec: $4.62 billion annually (2005–07); approximately $1.78 billion was allocated to financial costs and $2.84 billion to human costs. - Sectors such as mining, construction, forest products and transportation stand out as having significant costs associated with workplace incidents. - Noise exposure is the cause of the highest average costs per case, traditional indicators such as frequency and duration may not fully capture the true impact of such exposures.	The study underscores the financial burden of workplace incidents on employers and the healthcare system, highlighting the human costs and sectors needing increased prevention effort.
10	Bađun [15]	Croacia	2015	Secondary: official sources	Willingness to pay	Medical costs, Physical disability compensation, Administrative costs Legal costs disability pensions Costs arising from compliance with health and safety regulations Productivity losses, Costs arising from compliance with health and safety regulations.	HRK	Only the financial costs of the government (297) and employers (604.6) in 2015 of around HRK 900 million (0.3% of GDP).	Employers invest significant funds in preventing occupational injuries and illnesses, yet financial costs remain high. This situation underscores the need for the government to evaluate the effectiveness of the existing legal framework for occupational safety and health.
11	Rognstad [16]	Norway	1990	Secondary: Official sources	Market price model (gross output or human capital approach)	Material damage. Sick pay Rehabilitation, Health insurance, Medical treatment. The individual loss of income Medical cost Time off work. Time lost by colleagues and management Replacement of the worker.	NOK	Annual costs around NOK 40 billion (6.4% of GDP). Public sector covers 80% of costs, while companies primarily handle accident and disability claims. Individuals bear the costs of lost income due to permanent incapacity.	- The market model is inadequate for management decisions on security investments. - Reform is needed to shift costs from public to business sectors, encouraging investment in safety management to reduce workplace accidents - A clear definition of occupational diseases is needed. - Importance of proactive safety measures. - Need for better costing models and the importance of effective safety management to prevent occupational accidents and diseases in the workplace.
12	Shalini [17]	Mauritius	July 2002–June 2003	Mixed	Human capital	Medical cost (transfer to hospital + admission to hospital + medical staff + other medical resources) Occupational Health and Safety Investigation costs Loss of income because of days of absence from work Loss of income because of permanent disability death’s loss of productivity	RS	Total accident costs: 84.005.271,60 RS. Highest costs from productivity loss due to incapacity or death.	Interviews indicate a need for heightened health and safety awareness among employees and employers to prevent accidents
13	Miller and Galbraith [18]	United States	1990	Secondary: official sources	Willigness to pay	Medical cost Insurance Administrative and legal costs Wage and household work Workplace disruption Quality of life	USD	Workplace injuries cost society approximately $140 billion annually, including $17 billion in medical services and $60 billion in productivity losses.	Focus on targeting workplace injury intervention programmes, especially concerning costly traffic accidents at work.
14	Leigh *et al*. [19]	United States	1993	Secondary: official sources	Willingness to pay	Medical cost Wage loss Household production loss Pain and suffering loss	USD	- Highest average costs were in (cost per worker): taxicabs, bituminous coal and lignite mining, logging, crushed stone, oil field services, water transportation services, sand and gravel, and trucking. - Industries high on the total-cost list were trucking, eating and drinking places, hospitals, grocery stores, nursing homes, motor vehicles, and department stores. - Industries at the bottom of the cost-per-worker list included legal services, security brokers, mortgage bankers, security exchanges, and labour union offices.	Focus on high-cost sectors to enhance occupational safety and health performance.
15	Leigh *et al*. [20]	United States (California)	1992	Secondary: official sources	Capital human	Medical Cost Medical Administration Cost Lost Home Production, Lost Earnings Lost Fringe Benefits	USD	- Total estimated costs in California: $20.7 billion (1992), with injuries accounting for $17.8 billion (86%) and illnesses for $2.9 billion (14%) - Direct costs (34%) and indirect costs (66%) significantly impact finances. - Injuries cost $17.8 billion (86%) and diseases $2.9 billion (14%).	Costs comparable to total cancer costs in California; workers' compensation covers less than half of occupational injury costs.

## Discussion

The synthesis offers a comprehensive overview of the literature, delineating between determinants and costs. It facilitates the identification of consensus, contradictions, and limitations.

### Determinant studies

The literature on determinants has been investigated using two types of sources: Primary sources [6, 7] and secondary sources [8–10].

Primary sources (surveys, questionnaires and/or interviews) require a significant investment of time and resources on the part of the researchers, as the reliability of the study depends on adherence to very strict protocols that must be followed. Moreover, the need for these studies to be evaluated by an ethics committee is becoming increasingly common, given that they deal with data sensitive to the privacy of the participants. Furthermore, the authors of the articles that have used a survey emphasize that the interpretation of the results requires caution due to the presence of a possible selection bias and the use of a self-administered questionnaires. Additionally, the samples must focus on a more specific population than those using official data, and therefore, the results may be even less extrapolable and comparable. However, it allows for the measurement of variables that cannot be observed as comprehensively and directly way as with official databases, such as alcohol consumption.

The second option commonly chosen by researchers is to utilize secondary sources, typically official records. The primary advantage of secondary sources is that they tend to be comprehensive, especially when combined to gather as much information as possible. However, this does not eliminate biases in sampling or data recording in official records. Furthermore, these sources often lag in publication and sometimes lack availability of all variables within the study range.

This review has identified the two main approaches in studies on determinants of occupational accidents [7]:

Firstly, the occupational approach, which includes organizational-level analyses. The resources and organizational structure of financial companies are considered significant predictors of accidents and illnesses. Similarly, this approach includes macroeconomic factors that impact the company and its accidents, such as the economic cycle. A considerable portion of the studies reviewed in this review has attempted to explain whether there is such a relationship between accidents and the economic cycle. Economic fluctuations affect many factors related to occupational safety. These authors emphasize that, although many studies of this kind have been conducted, there is still insufficient scientific evidence to confirm the relationship between economic situation and the rate of occupational accidents [8, 10].

Secondly, the human error theory includes studies based on personal characteristics or moral attitudes towards worker safety, among other factors. This knowledge is important as it demonstrates that preventive measures related to these factors can be applied across all professional sectors, not just limited to specific industries. In fact, the authors converge on a common point: the need to devise and plan occupational risk prevention campaigns tailored to workers exposed to greater risks or those more vulnerable to occupational injuries.

Regarding the limitations of this type of study, they primarily stem from the fact that this literature focuses on a problem, occupational accidents, within a specific location and time period. Therefore, it is possible that the findings may not correspond to patterns observed in other countries or at different times.

Also, studies that aim to assess the business cycle need to include control variables related to work organization and the worker, as job fatigue or firm size which cannot be directly measured at the macro level. However, these variables are often represented by explanatory constructs, and gaps or biases may arise between the constructs and the proxies. At times, the identified associations do not provide sufficient evidence to draw firm conclusions about the mechanisms involved and only allow speculation regarding the reasons behind the relationships between economic situation and occupational accidents [10].

There are also other limitations such as the configuration or definition of variables. For example, in incidence rates, the increasing prevalence of part-time employment would lead to a downward bias in injury rates if the denominator of such a series were simply based on the number of employed persons. Similarly, when comparing productive sectors, divergences could arise due to differences in occupational risk structures.

### Gender studies

In articles that have focused on gender as a differentiating and determining factor in occupational accidents, different trends have been observed depending on the gender evaluated. Thus, gender is understood as a predictive and explanatory factor for accidents [6, 7]. Likewise, studies that omit its consideration conclude that it is important to account for it alongside other worker characteristics [7].

It is essential to clarify that the terms ‘gender’ and ‘sex’ are often used interchangeably in the literature. However, gender and sex are not the same. According to the WHO, ‘sex’ refers to the biological and physiological attributes that define men and women, while ‘gender’ refers to the socially constructed roles, behaviours, activities, and attributes that a given culture considers appropriate for men and women.

### Cost driver studies

The primary source of cost studies on accidents is secondary data, particularly government sources (accident surveys, compensation records, or jury verdicts). Through these sources, authors make a series of estimations to calculate the total cost of accidents. However, these sources may exclude certain factors, and most authors conclude that they often underestimate accident figures [11–13]. Similarly, some cost components, such as compensation payments, may be overestimated. In fact, some authors combine quantitative approaches with qualitative research methods to calculate the true economic or social costs of workplace accidents. In these studies, the three major categories of costs associated with occupational injuries and illnesses are typically recognized: direct costs, indirect costs, and quality-of-life costs [11]. Direct costs generally include payments for hospital services, medical services, and related expenses [13]; indirect costs refer to the productivity losses due to worker injuries [11]; and quality-of-life costs relate to the pain and suffering experienced by the worker and their family. When any of these categories are not accounted for, it is considered a significant limitation in cost studies [13].

Different economic models are increasingly utilized in the literature to calculate these costs:

The Friction Cost method limits productivity losses to the friction period, which refers to the time required to restore productivity to its pre-accident level [14, 15]. However, while this method is mentioned in the literature, it was not employed in any of the reviewed articles.

The Market Pricing model is a standard approach in calculating accident costs [16]. This method only considers direct and indirect costs, overlooking the intrinsic value of life, which cannot be monetarily measured. As a result, the costs are often underestimated. Despite this limitation, the market pricing model offers advantages such as straightforward documentation of calculations, avoidance of individual risk preferences, and awareness of the risks faced. Furthermore, this model is unaffected by the issue of incomplete or asymmetric information.

The Human Capital method similarly categorizes costs into direct ones. In the context of occupational injuries and illnesses, the Human Capital model calculates the economic impact by considering the potential loss of productivity and income resulting from these incidents. However, this method has been criticized for several reasons, including its failure to account for costs incurred by individuals who do not earn a salary, the underestimation of costs for women and minorities—whose wages are often lower due to discrimination- and its omission of substitution effects, inability to account for human capital investment, limited scope of analysis, neglect of the social perspective, and the sensitivity of its underlying assumptions [17].

The Human Capital approach is often supplemented by other methods to gain a more comprehensive understanding of the true costs and effects of injuries and illnesses. For instance:

Willingness to Pay is the most modern approach to calculating the cost of damage. It is based on estimating the amount of money that an individual or society is willing to pay, or accept, in exchange for reduced exposure to the risk of injury, illness, or death. This method is the most frequently chosen by authors to determine the value of a life [12, 17, 18].

The Quality-Adjusted Life Year or Disability-Adjusted Life Year (DALY) method evaluates changes in an individual’s health status. The Jury Awards method is gaining popularity in studies in the United States, based on the assumption that the costs of decreased quality of life can be estimated as the difference between the compensations awarded by a jury and the financial costs claimed by the victim [11].

At times, researchers combine various methods to obtain the most accurate estimates [15]. However, these approaches are criticized for their subjectivity in valuation. Assigning a monetary value to human life, health, and well-being can be ethically and morally controversial, as it may not fully reflect intangible costs, such as pain, suffering, and reduced quality of life.

Overall, the choice of method to apply depends on the availability of data and the purpose for estimating costs. A complete estimation of the costs of occupational injuries and illnesses is not always necessary. It is more useful to use a cost estimation method that produces results sufficiently reliable to serve as a basis for decision-making. Particularly, it is important to avoid double-counting when aggregating costs [15].

The main limitations of cost studies largely stem from unestimated cost components that and methodological decisions and limitations that can influence the estimates. These include, among others:

- The costs borne by society are not simply the sum of costs among various stakeholders. These assumptions may not necessarily be accurate, leading to either underestimation or overestimation of costs.
- The cost estimation methodology used in the study often relies on assumptions and the extrapolation of national data to reflect specific circumstances, which can introduce uncertainties into the estimates. Therefore, such studies are hardly extrapolatable and comparable.

In certain cost studies, there is a trend towards integrating cost studies with those on determinants [14, 19]. Specifically, costs are analysed depending on employee characteristics (age, gender, nationality), occupations, sectors, or specific injuries. However, the relationship between accident determinants and costs is not exhaustively studied.

### Limitations of the review process

Firstly, it is possible that some studies may have been excluded from the findings due to bias in setting selection criteria, using specific databases, and excluding of articles not written in English [21]. Additionally, variability in the terminology used to describe occupational accidents may have led to the omission of certain studies. Publication bias may also have played a role, as there is a tendency to publish studies with significant findings, while those with less impactful or negative findings remain unpublished [22]. However, the secondary review helped mitigate these limitations, and articles from non-indexed journals were considered. Finally, a meta-analysis was not possible due to the heterogeneity of the included studies. Despite these limitations, the main strength of this work lies in the use of a systematic and structured search methodology, as well as a specific approach to developing the narrative synthesis.

Comprehending the determinants and costs of injuries is vital for policymakers, employers, and researchers to effectively manage occupational safety and health risks, and to allocate resources efficiently for preventing workplace injuries and mitigating associated costs. However, many risks and costs remain insufficiently understood, resulting in their underestimation. Moreover, the review presented in this article offers key insights within these domains. Specifically, it highlights the primary perspectives and models employed in current research, which may shape the quality of future studies. The findings not only demonstrate advancements in occupational injury science but also reveal its limitations and areas for improvement. As a result, they suggest potential directions for further research, such as conducting studies that integrate both cost and determinant perspectives and producing more internationally and cross-sectorally comparable economic studies and evaluations.

Conflict of interest: None declared.

## Data Availability

The data supporting the findings of this study are available within the article.

## Submission declaration statement

All authors state that this article is not under consideration elsewhere or have not already been published, and that it will not be submitted for publication elsewhere without the agreement of the Managing Editor.

## Information on rights

Authors state that they observe the usual rules and practices regarding the reproduction of copyright material in their articles, assuming responsibility for obtaining permission where appropriate.
Key points

- Fifteen studies were ultimately included in this review; this research included results on determinants and cost-determinant studies.
- The included studies examined the relationship between accidents and personal characteristics or related factors of workers, as well as with economic cycles and organisational structure.
- The inclusion of the three types of costs—direct, indirect, and quality of life—is necessary for an accurate and appropriate economic valuation of occupational accidents.
- The sex of the worker is identified as a distinct and determining factor in occupational accidents.
